# 3D Printable Composite Polymer Electrolytes: Influence of SiO_2_ Nanoparticles on 3D-Printability

**DOI:** 10.3390/nano12111859

**Published:** 2022-05-29

**Authors:** Zviadi Katcharava, Anja Marinow, Rajesh Bhandary, Wolfgang H. Binder

**Affiliations:** Macromolecular Chemistry, Division of Technical and Macromolecular Chemistry, Faculty of Natural Sciences II (Chemistry, Physics, Mathematics), Institute of Chemistry, Martin Luther University Halle-Wittenberg, von-Danckelmann-Platz 4, D-06120 Halle, Germany; zviadi.katcharava@chemie.uni-halle.de (Z.K.); anja.marinow@chemie.uni-halle.de (A.M.); rajesh.bhandary@chemie.uni-halle.de (R.B.)

**Keywords:** polymer composite electrolyte, 3D-printing, silica nanoparticles, supramolecular polymers

## Abstract

We here demonstrate the preparation of composite polymer electrolytes (CPEs) for Li-ion batteries, applicable for 3D printing process via fused deposition modeling. The prepared composites consist of modified poly(ethylene glycol) (PEG), lithium bis(trifluoromethylsulfonyl)imide (LiTFSI) and SiO_2_-based nanofillers. PEG was successfully end group modified yielding telechelic PEG containing either ureidopyrimidone (UPy) or barbiturate moieties, capable to form supramolecular networks via hydrogen bonds, thus introducing self-healing to the electrolyte system. Silica nanoparticles (NPs) were used as a filler for further adjustment of mechanical properties of the electrolyte to enable 3D-printability. The surface functionalization of the NPs with either ionic liquid (IL) or hydrophobic alkyl chains is expected to lead to an improved dispersion of the NPs within the polymer matrix. Composites with different content of NPs (5%, 10%, 15%) and LiTFSI salt (EO/Li^+^ = 5, 10, 20) were analyzed via rheology for a better understanding of 3D printability, and via Broadband Dielectric Spectroscopy (BDS) for checking their ionic conductivity. The composite electrolyte PEG 1500 UPy_2_/LiTFSI (EO:Li 5:1) mixed with 15% NP-IL was successfully 3D printed, revealing its suitability for application as printable composite electrolytes.

## 1. Introduction

Since their commercialization in 1990s rechargeable lithium ion batteries (LIBs) are considered the most promising candidates as alternative clean energy sources beyond fossil fuels. Although LIBs exhibit several advantages such as high energy density, low self-discharge and long cycle life, the potential safety issues connected to the wide use of volatile, leachable and highly flammable organic liquid electrolytes, present main bottlenecks hampering their further development. Solid polymer electrolytes (SPEs) displaying low flammability, enhanced electrochemical performance, good processability and flexibility, have the potential to overcome the limitations associated with liquid electrolytes tackling a route towards safe next-generation high energy density batteries [1,2]. Although in the meantime various polymeric materials such as polycarbonates, poly(methacrylate)s, poly(acrylonitrile)s or poly(ionic liquid)s have been investigated as SPEs, poly(ethylene glycol) (PEG) is still the most investigated polymer in this area, due to its relatively low melting- and glass transition temperature and its ability to dissolve large amounts of lithium salts and actively participate in Li-ion transport [1,2,3,4,5]. Nevertheless, low room temperature ionic conductivity (10^−8^–10^−5^ S/cm) as well as moderate mechanical integrity of SPEs still makes the development of alternative electrolyte materials desirable. 

While PEG with molecular weights above 10 kDa displays an insufficient Li-ion conductivity [4,5] one of the promising approaches is the incorporation of inorganic (nano)particles into SPEs, where the synergy of inorganic and organic materials leads to an improvement of material properties and conductivity, such as Li-ion transport. In the meantime, various hybrid- (inorganically and organically modified nanocomposites), as well as so called composite electrolytes (CPE, physical mixing of components) have been investigated [1,4,5,6,7,8,9]. It should be mentioned, that in literature blurred borders between the terms “composite” and “hybrid” electrolyte can be observed. Already in early 1980s Weston and Steele have shown that the incorporation of α-alumina particles into PEG containing LiClO_4_ as conducting salt in up to 10 vol% significantly increased the mechanical stability of the prepared composite electrolyte, whereas an influence on the conductivity was neglectable [10]. In the past decades various chemically inert inorganic oxide (nano)particles (e.g., SiO_2_, Al_2_O_3_, TiO_2_, ZrO_2,_ ZnO) have been evaluated as ceramic fillers for composite polymeric electrolytes, resulting not only in an increase in mechanical integrity but also enhancing ionic conductivity and lithium ion transport trough the electrolyte [1,6,9,11]. Furthermore, Archer and coworker achieved enhanced dispersivity of SiO_2_ nanoparticles in polycarbonate by covalently tethering ionic liquids (ILs) on the surface of SiO_2_ nanoparticles. The resulting composite electrolytes revealed both, increased ion conductivity and mechanical stability, enhancing the performance and lifetime of the battery [12]. 

In addition to electrochemical performance and safety, also life-time and processability plays a crucial role, lowering the total costs of next generation LIBs. For example, incorporation of self-healing polymers, which are able to autonomously repair battery components after damage e. g. mechanical defects due to volume expansion or cracking, chemical- or thermally-induced degradation, can significantly prolong the lifetime of a battery [13,14]. One promising approach to introduce self-healing functionality is the use of reversible-dynamic bonds such as hydrogen-bonds, [15,16] capable to respond to internal or external changes, thus compensating damage caused by volume changes or mechanical stress during charging/discharging. Thus, the attachment of strong and reversible hydrogen-bonding moieties, such as ureidopyrimidone (UPy) groups to polymer backbones (e.g., PEG or poly(acrylic acid), PAA) leads to formation of dynamic supramolecular networks via hydrogen-bonding, enabling the use of polymers as self-healing binders for high-performance silicon nanoparticle (SiNP) anodes and thus compensating for volume changes during charging/discharging [17,18]. In order to improve the processability of solid phase electrolytes as well as battery components in general, significant efforts are made towards developing of 3D-printable materials [19,20]. 3D-printing techniques provide an opportunity for feasible manufacturing with high complexity and fine features [21,22,23]. Fused deposition modeling (FDM) based on layer-by-layer deposition of thermoplastic filaments, is one of the most popular methods used in both industry and academia. Basically, molten polymers are extruded trough a heated nozzle and deposited onto a substrate, where upon cooling it turns back into a solid. However, despite its popularity FDM has not been commonly used for battery components especially for electrolyte materials due to the limited conductivity of thermoplastic filaments [24]. Recently, reports indicated that the optimization of the conductive materials is a successful approach to overcome this conductivity barrier [25]. Furthermore, Dupont and coworkers recently used an additive manufacturing technology to prepare a printable polyethylene oxide/lithium bis(trifluoromethanesulfonyl)imide (PEO/LiTFSI) filament which can be subsequently fed into an FDM 3D printer [26]. Thus, development of novel materials is needed to make current 3D-techniques capable to fulfill requirements for battery production [27]. 

Recently, we presented a profound investigation on the factors influencing the printability of SPE electrolytes containing quadrupole (UPy)-hydrogen bonds and lithium salts [28]. We have shown that both, the self-healing property and the printability of the telechelic UPy-PEO/PPO-UPy polymer can be tuned not only by temperature, an already known tool for adjusting melt-rheology, but also by variation of the salt content, which consequently influences the crystallinity of the polymer. Herein, we want to go a step further and present the preparation of 3D-printable composite polymer electrolytes, consisting of telechelic PEG bearing hydrogen bonding moieties (UPy and barbiturate, B), a corresponding lithium salt and SiO_2_ nanoparticles (NPs) as inorganic fillers. In order to investigate the influence of size and dispersivity as well as the nature of the used nanoparticles on the properties of the resulting composite electrolyte two different types of SiO_2_ NPs have been evaluated and additionally the surface of the particles was modified by covalent tethering of either ionic liquid or hydrophobic alkyl chain. Both, the prepared modified NPs and the corresponding composites have been profoundly characterized, and the influence of the type and content of NPs on the ion transport properties and rheological behavior of the CPEs have been studied. By adjusting the NPs/salt ratio printable electrolytes with good conductivity can be achieved. 

## 2. Materials and Methods

### 2.1. Chemicals

PEG 1500, PEG 8000, LUDOX^®^ SM colloidal silica, silica nanopowder (diameter 12 nm, surface area 175–225 m^2^/g), N-methylpyrrolidine, hexamethylene diisocyanate, methanesulfonyl chloride and sodium azide were purchased form MilliporeSigma (Darmstad, Germany). 2-Amino-4-hydroxy-6-methylpyrimidine was received form TCI (Eschborn, Germany). (3-chloropropyl)trimethoxysilane and triethylamine were purchased form Alfa Aesar (Kandel, Germany). (Dodecyl)dimethylsilane was obtained from Acros Organics (Geel, Belgium). Triphenylphosphine was purchased form Roth. Lithium bis(trifluoromethylsulfonyl)imide (LiTFSI) was received form IoLiTec (Eschborn, Germany).

### 2.2. Instrumentation

FT-IR spectrum was recorded using attenuated total reflection technique on VERTEX 70 v FT-IR Spectrometer (Bruker) equipped with the golden gate diamond ATR unit. Measurements were conducted at room temperature and covered spectral range was from 550 cm^−1^ to 4000 cm^−1^.

Thermogravimetric analysis (TGA) was conducted on Netzsch TG 209 F3. 5–10 mg samples were placed in alumina crucibles and heated under inert atmosphere with the heating rate of 10 K/min. 

Differential scanning calorimetry (DSC) measurements were carried out on a Netzsch DSC 204 F1. Samples were dried before measurement in vacuum at 80 °C and placed in aluminum pans, with measurements being conducted under an atmosphere of nitrogen. The thermal history was removed by heating samples up to 100 °C. Cooling was carried out down to −20 °C with the rate 5 K/min. Heating curves were recorded up to 170 °C with the heating rate 5 K/min. 

Rheology measurements were conducted on an Anton Paar MCR-101 DSO rheometer equipped with parallel plate-plate geometry (diameter of 8 mm). Samples were dried at 80 °C in the vacuum for 24 h and shear rate vs. viscosity measurements were performed at different temperatures. 

A Broadband Dielectric Spectroscopy (BDS) Novocontrol “Alpha analyzer” was used for investigating ionic conductivity. Polymer samples were placed in a cell containing two brass electrodes with the dimension of the sample space of 20 mm diameter and 2.5 mm thickness and the cell was placed in a cryostat with a constant flow of dry nitrogen. Ionic conductivity was recorded in the frequency range 1–10^6^ Hz. 

3D printing was performed using a RegenHU 3D Discovery equipped with a heatable tank and an extrusion printing head. A needle with the size of 0.33 mm was connected to the printing head and a pressure of 0.15 MPa was applied to generate a flow of molten polymer to the printing head. The desired shape was constructed using BioCAD™ program; fused deposition modeling (FMD) was performed by directly printing on glass surfaces. All samples were dried in the vacuum at 80° for 48 h prior the printing. 

## 3. Results and Discussions

### 3.1. Modification of PEG Polymers

**Figure 1:**
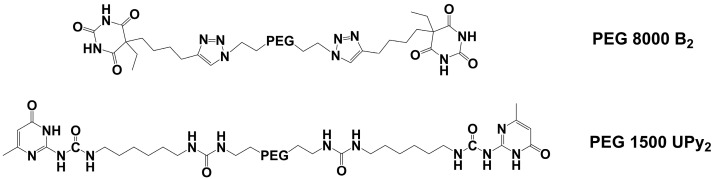
Chemical structures of **PEG 8000 B_2_** and **PEG 1500 UPy_2_**.

PEG 1500 and PEG 8000 were end group modified according to already known procedures [20,29,30], in order to obtain telechelic polymers containing hydrogen bonding moieties, either the barbiturate in **PEG 8000 B_2_** or the ureidopyrimidone in **PEG 1500 UPy_2_**. Obtained polymer structures are schematically presented in the Figure 1. Purity of the products were analyzed using ^1^H-NMR (Supplementary Materials Figures S1 and S2). The two hydrogen bonding samples display a largely different strength: while barbiturate forms (weakly bonded) H-bonded clusters [31,32,33,34], the UPy group forms (strong) dimeric-assemblies via their quadrupole-type hydrogen bonds [16,29].

### 3.2. Modification of Nanoparticles

#### 3.2.1. Modification of LUDOX^®^ SM SiO_2_ NPs by Using Ionic Liquid Groups

**Figure 2:**
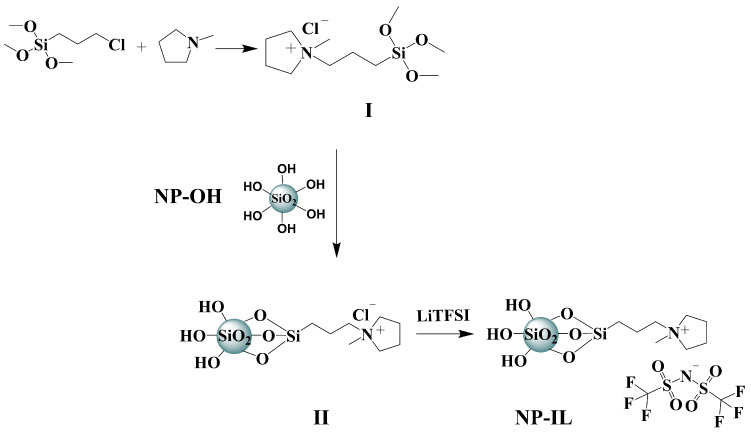
Surface modification of Ludox-sm^®^ 30 with ionic liquid groups (**NP–IL**).

Modification of silica particles was adopted from previously reported procedures [35] and the preparation route is schematically presented in Figure 2. Initially N-[3-(trimethoxysilyl)propyl]-N-methylpyrrolidinium chloride (**I**) was synthesized from N-methylpyrrolidine and (3-chloropropyl)trimethoxysilane (detailed description of the synthesis procedure can be found in the supporting information). In a typical modification of silica nanoparticles Ludox-sm^®^ (3 g) was diluted using deionized water (100 g) and 0.7 g (2.5 mmol) of (3-chloropropyl)trimethoxysilane were added to the solution. The mixture was kept stirring at 80 °C for 24 h. Solutions containing the modified nanoparticles (**II**) were concentrated using a rotary evaporator and precipitated into acetone. NPs were collected using centrifugation and washed with acetone three more times. For obtaining the final modified particles with TFSI anion a simple anion exchange reaction was conducted via the following procedure: **II** was dissolved in 35 mL deionized water and mixed with 10 mL of a solution containing 1 g (3.5 mmol) LiTFSI. The mixture was kept stirring for 8 h, after which the final product (**NP–IL**) was collected via centrifugation and washed several times with deionized water. **NP–ILs** were dried under vacuum at 70 °C and stored in a desiccator over P_2_O_5_.

#### 3.2.2. Modification of Silica Nanopowder

**Figure 3:**
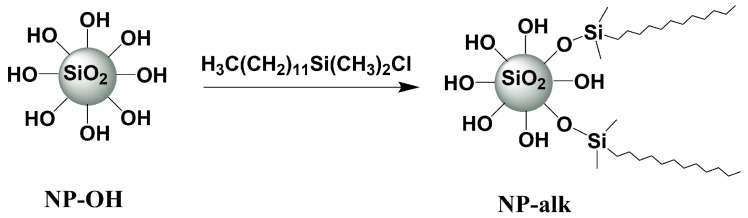
Surface modification of silica nanopowder using alkyl groups (**NP–alk**).

Nanopowder was modified with (dodecyl)dimethylsilane groups which is schematically presented in Figure 3. 1 g of previously dried (170 °C for 72 h under high vacuum) nanopowder was dispersed in 50 mL of dry DCM for 60 min. Chloro(dodecyl)dimethylsilane (3.0 mL, 10 mmol) and pyridine (0.9 mL, 10 mmol) were added to the suspension and stirred for 8 h. The so modified nanoparticles were collected using centrifugation, re-dispersed in cold dry DCM and collected again. This washing procedure was repeated three times and the product (**NP–alk**) was dried in high vacuum at 50 °C for 24 h.

#### 3.2.3. Characterization of Modified Nanoparticles

**Figure 4:**
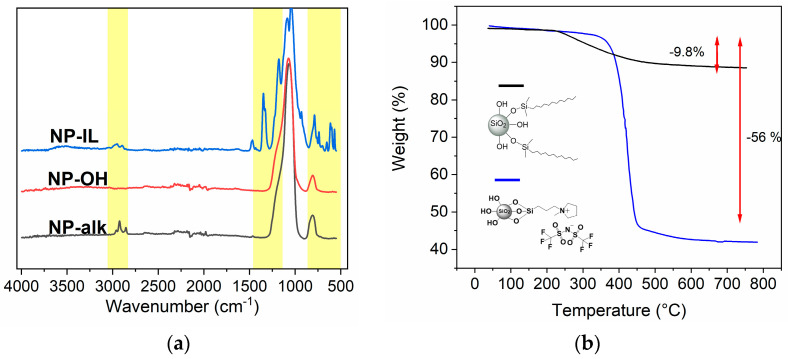
(**a**) **FT–IR** spectra of nanoparticles **NP–IL** (blue), **NP–OH** (red) and **NP–alk** (black); (**b**) TGA thermograms of **NP–Alk** (black) and **NP–IL** (blue).

Modified nanoparticles were analyzed using FT-IR and TGA. Figure 4a shows the IR spectra of **NP–IL**, **NP–OH** and **NP–alk** for comparison. **NP-OH** shows a strong absorption peak at 1055 cm^−1^ corresponding to the stretching vibration of the Si-O-Si bond. After modification the appearance of new signals can be observed. **NP–IL** shows characteristic signals of the alkane C-H stretching vibration at wavenumbers of 2800–3000 cm^−1^. Furthermore, bands from the TFSI anion are presented: SO_2_ stretching at 1348 cm^−1^, CF_3_ stretching at 1179 cm^−1^_,_ SNS stretching at 1086 cm^−1^, C-S stretching at 788 cm^−1^. [36,37] **NP–alk** exhibits only the stretching vibration of the alkene C-H at 2800–3000 cm^−1^. Results of the thermogravimetric analysis are presented in Figure 4b. Modified nanoparticles showed a weight loss at higher temperatures originating from the decomposition of the organic modifiers at surfaces, indicating a successful functionalization. **NP–IL** displayed a thermal stability of up to 350 °C, which is in accordance with the results obtained for the IL-functionalized NPs known from literature, [35] whereas the thermal stability of **NP–alk** is significantly lower (up to 250 °C). The size of the NPs (ranging from 10–120 nm) was determined via DLS and TEM measurements and the successful surface modification was additionally confirmed via solution ^1^H-NMR and solid CP/MAS ^29^Si-NMR (see Supplementary Materials).

### 3.3. 3D Printability of Composite Electrolytes via Rheology

The desired composite electrolytes were prepared by mixing of the modified telechelic polymers with lithium salt and nanoparticles and subsequently analyzed via melt rheology for their mechanical properties, in particular in view of their 3D printability. The addition of hydrogen-bonding end groups will strongly modify the thermal profile of the polymeric electrolytes, imposing a nonlinear melt-flow at temperatures, where the H-bonds are broken [38]. The nanoparticles in turn will also allow for a change of viscosity, so as to achieve both, adjustment of printability and an increased conductivity. Summary of samples compositions are given in the Table 1. Samples containing only polymer and lithium salt (samples **8**–**10**) were dissolved in dry ACN, thereafter the solvent was removed in an oven and then samples were completely dried in vacuum at 90 °C for 48 h. Samples containing the nanoparticles were ultrasonicated to ensure the proper de-agglomeration of the incorporated particles and then a similar drying procedure was used. For the preparation of samples **5**, **6** and **7** Ludox^®^ NPs were used and previously dried via freeze-drying. For comparison samples **18**, **19** and **20** were prepared with SiO_2_ nanopowder. 

**Figure 5:**
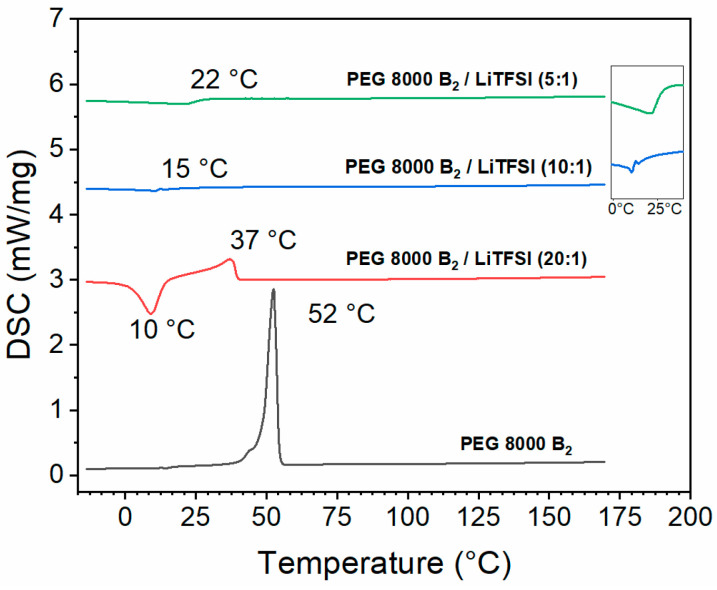
Differential scanning calorimetry (DSC) results of PEG 8000 B_2_ without any additives and PEG 8000 B_2_ with different amount of mixed LiTFSI (20:1(**10**), 10:1 (**9**), 5:1 (**8**)).

One of the common approaches to analyze the 3D printability of polymer is to investigate their behavior via melt rheology. Before the rheological measurements the thermal properties of polymers were investigated by using differential scanning calorimetry (DSC). PEG 1500 UPy_2_/LiTFSI was previously investigated showing no melting transition, indicating the absence of crystallinity [28]. For the **PEG 8000 B_2_** based compositions DSC curves were recorded form −15 °C to 170 °C and are presented in Figure 5. Pure **PEG 8000 B_2_** polymer displayed a crystalline behavior and a melting transition at 52 °C can be observed. PEG 8000 B_2_/LiTFSI (20:1) (**10**) showed crystalline behavior but the melting peak had shifted to a lower temperature at 37 °C. Furthermore, additional exothermic transitions can be seen in the DSC curves, which can be assigned to cold crystallization as a result of faster cooling rate before recording the heating curve, preventing full crystallization and quenching the sample into an amorphous phase [39]. For PEG 8000 B_2_/LiTFSI (10:1) (**9**) the melting peak has fully disappeared, indicating the absence of crystalline phases. PEG 8000 B_2_/LiTFSI (5:1) (**8**) showed a more distinct glass transition temperature at 22 °C and the absence of a melting peak, which is a characteristic behavior of amorphous structures. The increasing concentration of LiTFSI salt is supposedly hindering the formation of lamellar structures, thus preventing crystallization as previously reported in the literature for similar PEG/salt compositions [28,40]. With the obtained information about thermal transitions 3D printability was investigated above T_m_/T_g_ for all compositions.

The printing window for the used 3D printer geometry was studied using melt rheology measurements. Thus, the melt viscosity value thus should be in the range of 200 Pa×s to 2000 Pa×s. This is defined by the geometry of the printing needle together with the geometry and the temperature of the storage tank and the transfer line between. These rheological borders are well established, and have repeatedly been proven by us [20,28,38,41]. 

#### 3.3.1. Rheology Measurements of PEG 8000 B_2_ without Nanofillers

**Figure 6:**
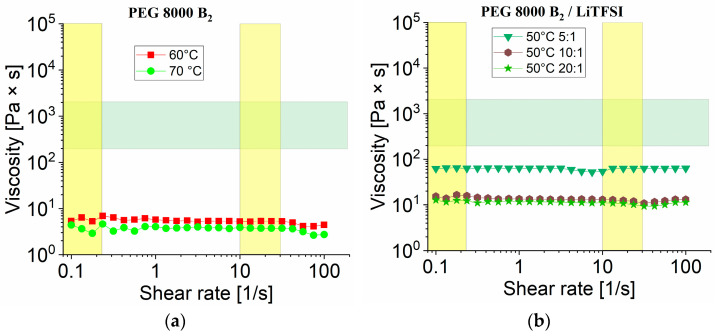
Rheology measurements viscosity vs. shear rate for (**a**) PEG 8000 B_2_ at 60 °C and 70 °C; (**b**) PEG 8000 B_2_ mixed with LiTFSI (EO/Li 5:1 (**8**), 10:1 (**9**), 20:1 (**10**)) at 50 °C.

Figure 6a shows the melt behavior of pure **PEG 8000 B_2_** above its melting point. The melt viscosity is far too low from the printing window (presented as cross section of green and yellow cross section), hence the sample is not applicable for 3D printing processes using fused deposition modeling. In addition, compositions containing **PEG 8000 B_2_** mixed with different ratios of LiTFSI (**8**, **9** and **10**) without nanofillers showed a lower viscosity than desired values at 50 °C given in Figure 6b. 

#### 3.3.2. Rheology Measurements of PEG 1500 UPy and PEG 8000 B_2_ with Nanofillers

**Figure 7:**
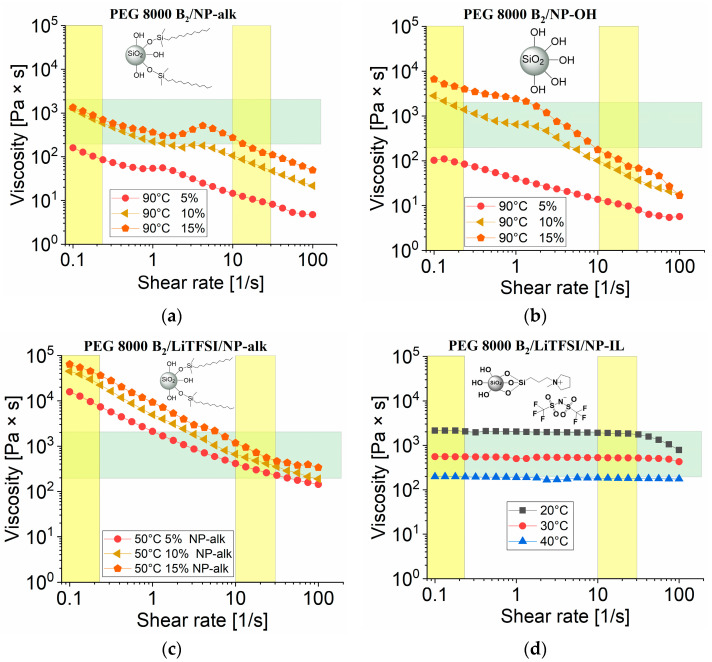
Rheology measurement of viscosity vs. shear rate for (**a**) PEG 8000 B_2_ mixed with **NP–alk** (5% (**15**), 10% (**16**), 15% (**17**)) at 90 °C; (**b**) PEG 8000 B_2_ mixed with **NP–OH** (5% (**18**), 10% (**19**), 15% (**20**)) at 90 °C; (**c**) PEG 8000 B_2_ mixed with LiTFSI (5:1) and **NP–alk** (5% (**11**), 10% (**12**), 15% (**13**)); (**d**) PEG 8000 B_2_ mixed with **NP–IL** (5% (**14**)).

In order to shift the viscosity into the printable range, NPs were incorporated in different amounts. Therefore **PEG 8000 B_2_** was investigated with varying amounts of nanofillers: **NP–alk** (**15–17**) and **NP–OH** (nanopowder, **18–20**). The rheology results are given in Figure 7. Samples **15**–**20** exhibit a drastic increase in viscosity even at higher temperatures. Sample **17** can be applicable for 3D printing as the viscosity vs shear rate is fitting into the desired range. Polymer composites in Figure 7b show shear thinning behavior which reduces viscosity below required values. Samples **11**–**13** (Figure 7c) containing LiTFSI with different ratios of the **NP**–**alk** also display a shear depended viscosity and will require more adjustment for performing 3D printing on our setup, as the viscosity values at low shear rate are too high to have the polymer melt flow from storage tank to printing head. Sample **14** (Figure 7d) containing additional **NP**–**IL** shows printability but at relatively low temperatures (20–40 °C) which can be problematic as printing carried out close to room temperature is less likely to keep the desired shape. 

**Figure 8:**
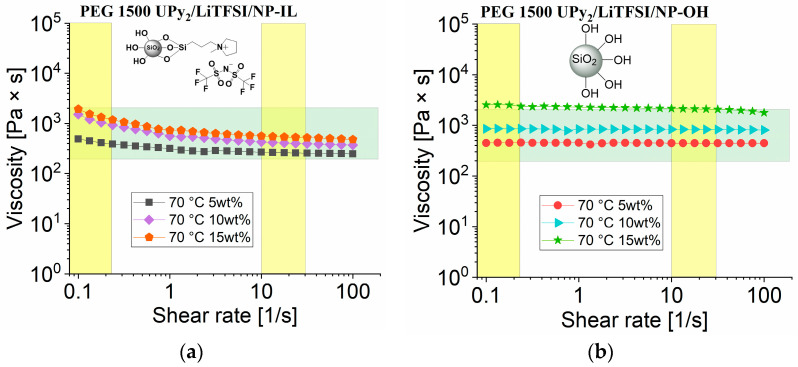
Rheology measurement of viscosity vs. shear rate for (**a**) PEG 1500 UPy_2_/LiTFSI mixed with **NP–IL** (5% (**2**), 10% (**3**), 15% (**4**)) at 70 °C; (**b**) PEG 1500 UPy_2_/LiTFSI mixed with **NP–OH** (5% (**5**), 10% (**6**), 15% (**7**)) at 70 °C.

Previously we reported that **PEG 1500 UPy_2_** as such is not suitable for 3D printing in our 3D-printer without the use of additional fillers [28]. Figure 8 represents the rheological measurements of PEG 1500 UPy_2_/LiTFSI now containing either **NP**–**IL** (**2**,**3**,**4**) or the **NP**–**OH** (**5**,**6**,**7**), showing increased viscosity and also linear behavior of shear vs viscosity. Thus, the presented composite materials exhibit promising properties suitable for 3D printing process. 

### 3.4. Conductivity

**Figure 9:**
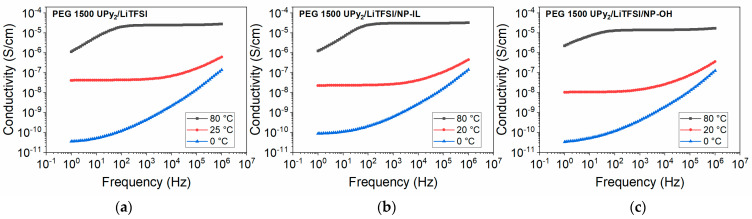
Frequency dependent ionic conductivity of (**a**) PEG 1500 UPy/LiTFSI mixture (EO:Li 5:1)(**1**); (**b**) PEG 1500 UPy/LiTFSI (EO/Li 5:1) mixed with 15 wt% **NP–IL** (**4**); (**c**) PEG 1500 UPy/LiTFSI (EO/Li 5:1) mixed with 15 wt% **NP–OH** (**7**).

DC conductivity was extracted from the DC plateau of the frequency vs. conductivity plots obtained from BDS measurements. The DC conductivities for the samples at the temperature 0 °C are not extracted (except PEG 8000 B_2_/LiTFSI mixture (EO:Li 10:1)) due to the considerable overlap of electrode polarization with the DC plateau. PEG 1500 UPy_2_ mixed with LiTFSI showed conductivities up to 2.8 × 10^−5^ S/cm at 80 °C (Figure 9a). The same sample with additional nanofillers (composition **4** and **7**) also exhibits conductivity in a similar range. **NP–OH** addition led to conductivity up to 1.7 × 10^−5^ S/cm at 80 °C, while addition of the surface modified NPs (**NP–IL**) led to slightly increased conductivities of up to 3.2 × 10^−5^ S/cm at 80 °C. This indicates that modification of the surface with the ionic groups may positively influence the conductivity of such composite electrolytes. 

**Figure 10:**
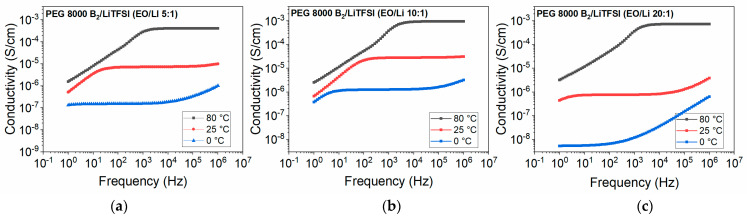
Frequency dependent ionic conductivity of (**a**) PEG 8000 B_2_/LiTFSI mixture (EO:Li 5:1) (**8**); (**b**) PEG 8000 B_2_/LiTFSI mixture (EO:Li 10:1) (**9**); (**c**) PEG 8000 B_2_/LiTFSI mixture (EO:Li 20:1) (**10**).

Similarly, conductivities of the **PEG 8000 B_2_** samples were obtained (Figure 10). PEG 8000 B_2_ was mixed with different ration of LiTFSI, where samples **8**, **9** and **10** now showed conductivities up to 10^−3^ S/cm at 80 °C. The conductivity of sample **8** (EO:Li 5:1) at 80 °C is slightly reduced compared to samples **9** and **10**, which can be due to the formation of ion aggregates in sample with higher concentration of salts and reduction of mobility of charged units [42].

### 3.5. 3D Printing of Nanocomposites

**Figure 11:**
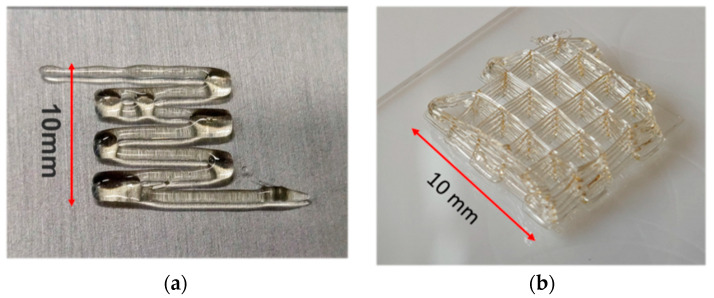
3D printing attempts of (**a**) PEG 1500 UPy_2_/LiTFSI (EO:Li 5:1)(**1**); (**b**) PEG 1500 UPy_2_/LiTFSI (EO:Li 5:1) mixed with 15% **NP–IL** (**4**).

3D printing was performed using fused deposition modeling (FDM) on a glass slide under ambient laboratory condition. The printer was equipped with temperature controllable storage tank and printing nozzle having needle (0.33 mm) attached. Sample **7** was dried in the vacuum at 90 °C for 48 h prior the experiment. During the FDM process, the storage tank temperature was set to 90 °C and the temperature of the printing head to 70 °C as the rheology profile at this temperature was fitting in the printing window. In Figure 11a the 3D printing attempt of PEG 1500 UPy_2_/LiTFSI (EO/Li 5:1) is shown, the sample had spread on the surface and could not sustain its shape. PEG 1500 UPy_2_/LiTFSI (EO:Li 5:1) mixed with 15% **NP–IL** (**4**) was 3D printed under same conditions and was photographed (Figure 11b). Composite **4** showed improved printability and mechanical properties, where six layers of extruded polymer were stacked to form the grid shape stable up to 1 h before water absorption (presumably by the hygroscopic LiTFSI in the electrolyte) caused noticeable structural changes as printing was carried out at ambient laboratory-conditions. 

## 4. Conclusions

We here have demonstrated the preparation of self-healing polymer composite electrolytes (consisting of modified PEG, LiTFSI and nanofillers) applicable for of 3D printing process via fused deposition modeling. PEG was successfully end group modified via UPy and barbiturate moieties for introducing hydrogen bonds, providing self-healing ability to the material. Silica nanoparticles were used as a filler for further improvement of the mechanical properties of the electrolyte. The NPs were surface modified with ionic liquid groups and short alkyl chains to control the interactions between the surfaces and the polymer in the compositions, thus adapting dispersivity and rheology of the composites. Samples with different content of NPs (5%, 10%, 15%) and LiTFSI salt (EO/Li^+^ = 5, 10, 20) were analyzed via rheology for better understanding of 3D printability and via BDS for checking their conductivity. The composite electrolyte PEG 1500 UPy_2_/LiTFSI (EO:Li 5:1) mixed with 15% NP-IL was successfully 3D printed into a grid shape, useful for further applications in multilayered structures and components. Moreover, the printing process did not have significant influence on the conductivity of the printed electrolyte. 

## Figures and Tables

**Table 1:** Electrolyte compositions prepared form PEG 1500 UPy_2_ and PEG 8000 B_2_.

Entry	Polymer	Molar EO/Li	Nanoparticles	(wt%) Nanoparticles
**1**	PEG 1500 UPy_2_	5:1	**-**	-
**2**	PEG 1500 UPy_2_	5:1	**NP–IL**	5
**3**	PEG 1500 UPy_2_	5:1	**NP–IL**	10
**4**	PEG 1500 UPy_2_	5:1	**NP–IL**	15
**5**	PEG 1500 UPy_2_	5:1	**NP–OH**	5
**6**	PEG 1500 UPy_2_	5:1	**NP–OH**	10
**7**	PEG 1500 UPy_2_	5:1	**NP–OH**	15
**8**	PEG 8000 B_2_	5:1	**-**	-
**9**	PEG 8000 B_2_	10:1	**-**	-
**10**	PEG 8000 B_2_	20:1	**-**	-
**11**	PEG 8000 B_2_	5:1	**NP–alk**	5
**12**	PEG 8000 B_2_	5:1	**NP–alk**	10
**13**	PEG 8000 B_2_	5:1	**NP–alk**	15
**14**	PEG 8000 B_2_	5:1	**NP–IL**	10
**15**	PEG 8000 B_2_	-	**NP–alk**	5
**16**	PEG 8000 B_2_	-	**NP–alk**	10
**17**	PEG 8000 B_2_	-	**NP–alk**	15
**18**	PEG 8000 B_2_	-	**NP–OH**	5
**19**	PEG 8000 B_2_	-	**NP–OH**	10
**20**	PEG 8000 B_2_	-	**NP–OH**	15

## Data Availability

The data that support findings of this study are available from the corresponding author upon reasonable request.

## Supplementary Materials

The following supporting information can be downloaded at: https://www.mdpi.com/article/10.3390/nano12111859/s1, Figure S1: ^1^H NMR of PEG 1500 UPY_2_ in CDCl_3_, Figure S2: ^1^H NMR of PEG 8000 B_2_ in CDCl_3_, Figure S3: ^1^H and ^13^C NMR N-[3-(trimethoxysilyl)propyl]-N-methylpyrrolidinium chloride, Figure S4: Rheology measurement of Viscosity vs. shear rate for PEG 1500 UPy_2_ mixed with NP-OH (5%, 10%, 15%) 60–80 °C, Figure S5: Rheology measurement of Viscosity vs. shear rate for PEG 1500 UPy2 mixed with NP-IL (5%, 10%, 15%) 50–70 °C, Figure S6: TEM image of NP-IL, Figure S7: DLS size distribution of NP-alk, Figure S8: ^1^H NMR of NP-IL in DMSO-d_6_, Figure S9: ^29^Si MAS NMR spectra of NP-IL, Figure S10. Frequency dependent ionic conductivity of (a) PEG 1500 UPy/LiTFSI (EO/Li 5:1) mixed with 15 wt% NP-IL (**4**) before and after FDM; (b) PEG 1500 UPy/LiTFSI (EO/Li 5:1) mixed with 15 wt% NP-OH (**7**) before and after FDM, Figure S11. (a) PEG 1500 UPy/LiTFSI (EO/Li 5:1) mixed with 15 wt% NP-OH (7); (b) cut sample; (c) Reconnected sample; (d) (e) (f) Stretch test after self-healing at 30 °C (in the vacuum) for 12 h.
