# Supplementary material for: 3D Printable Composite Polymer Electrolytes: Influence of SiO2 Nanoparticles on 3D-Printability
Source: Nanomaterials (Basel). 2022 May 29;12(11):1859. doi: 10.3390/nano12111859 (PMC9181955; doi:10.3390/nano12111859)
Supplement: Supplementary file 1 [file nanomaterials-12-01859-s001.zip › nanomaterials-1726981-supplementary.pdf]

nanomaterials

MDPI

Supplementary Information

3D Printable Composite Polymer Electrolytes: Influence of SiO_2 Nanoparticles on 3D-Printability

Zviadi Katcharava [1], Anja Marinow [1], Rajesh Bhandary [1] and Wolfgang H. Binder [1*]

Macromolecular Chemistry, Division of Technical and Macromolecular Chemistry, Faculty of Natural Sciences II (Chemistry, Physics, Mathematics), Institute of Chemistry, Martin Luther University Halle-Wittenberg, von-Danckelmann-Platz 4, D-06120 Halle, Germany; zviadi.katcharava@chemie.uni-halle.de (Z.K.); anja.marinow@chemie.uni-halle.de (A.M.); rajesh.bhandary@chemie.uni-halle.de (R.B.)

* Correspondence: wolfgang.binder@chemie.uni-halle.de

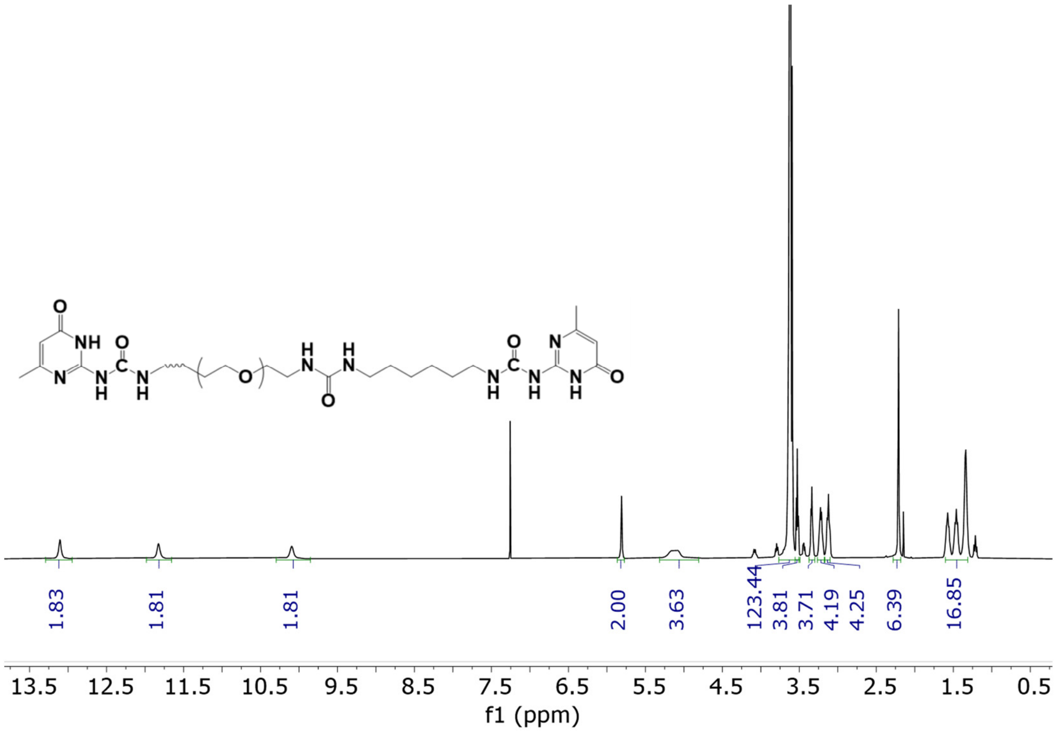

Figure S1. ^1H NMR of PEG 1500 UPY_2 in $CDCl_3$;

^1H NMR (400 MHz, $CDCl_3$) δ 13.11 (s, 2H), 11.83 (s, 2H), 10.09 (s, 2H), 5.81 (s, 2H), 5.38 – 4.78 (m, 4H), 3.62 (d, J = 3.0 Hz, 124H), 3.53 (t, J = 5.0 Hz, 4H), 3.34 (t, J = 5.0 Hz, 4H), 3.22 (d, J = 6.3 Hz, 4H), 3.12 (t, J = 7.1 Hz, 4H), 2.21 (s, 6H), 1.63 – 1.28 (m, 16H).

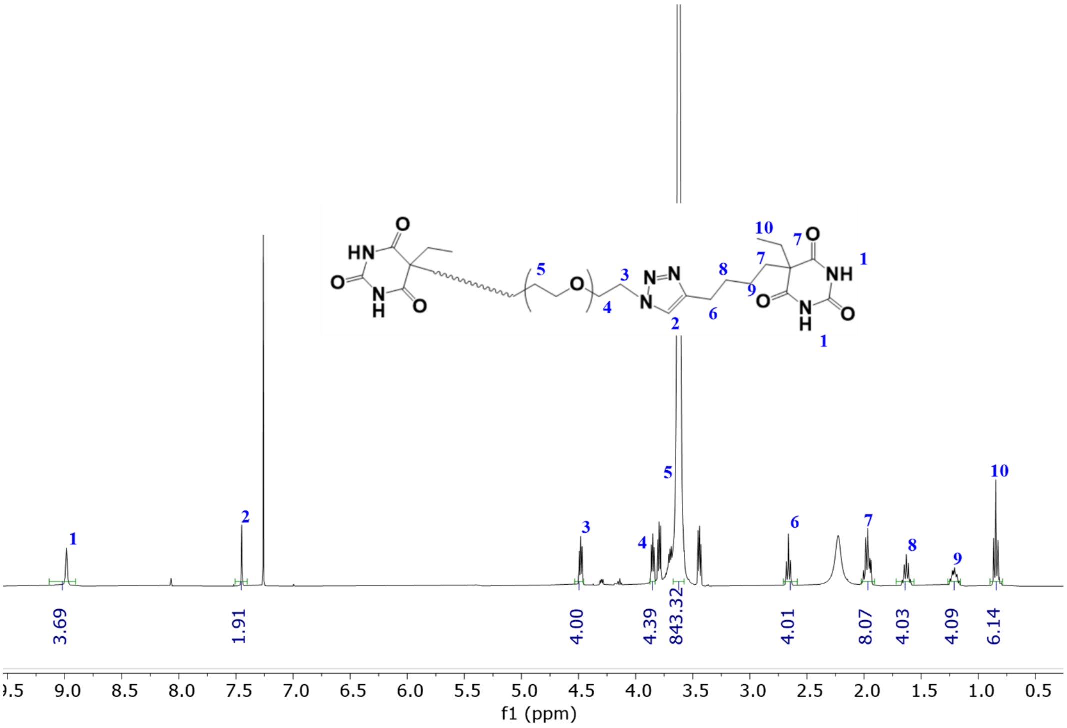

Figure S2. ^1H NMR of PEG 8000 B_2 in $CDCl_3$;

^1H NMR (400 MHz, $CDCl_3$) δ 8.98 (s, 4H), 7.45 (s, 2H), 4.48 (t, *J* = 4.4 Hz, 4H), 3.85 (t, *J* = 4.3 Hz, 4H), 3.62 (s, 840H), 2.66 (t, *J* = 7.2 Hz, 4H), 2.02 – 1.91 (m, 8H), 1.63 (p, *J* = 7.3 Hz, 4H), 1.27 – 1.16 (m, 4H), 0.85 (t, *J* = 7.4 Hz, 6H).

Synthesis of N-[3-(trimethoxysilyl)propyl]-N-methylpyrrolidinium chloride

N-methylpyrrolidinium (5 mL, 47 mmol) and (3-chloropropyl)trimethoxysilane (8.57 mL, 47 mmol) mixture were kept at 80 °C on stirring for 48 hours in 50 mL one neck flask. Color turned into red/orangish. Mixture were washed using diethyl ether 4 times 4x30ML and N-[3-(trimethoxysilyl)propyl]-N-methylpyrrolidinium chloride was dried in the vacuum. Yellowish/brownish solid product was obtained. Yield = 55%

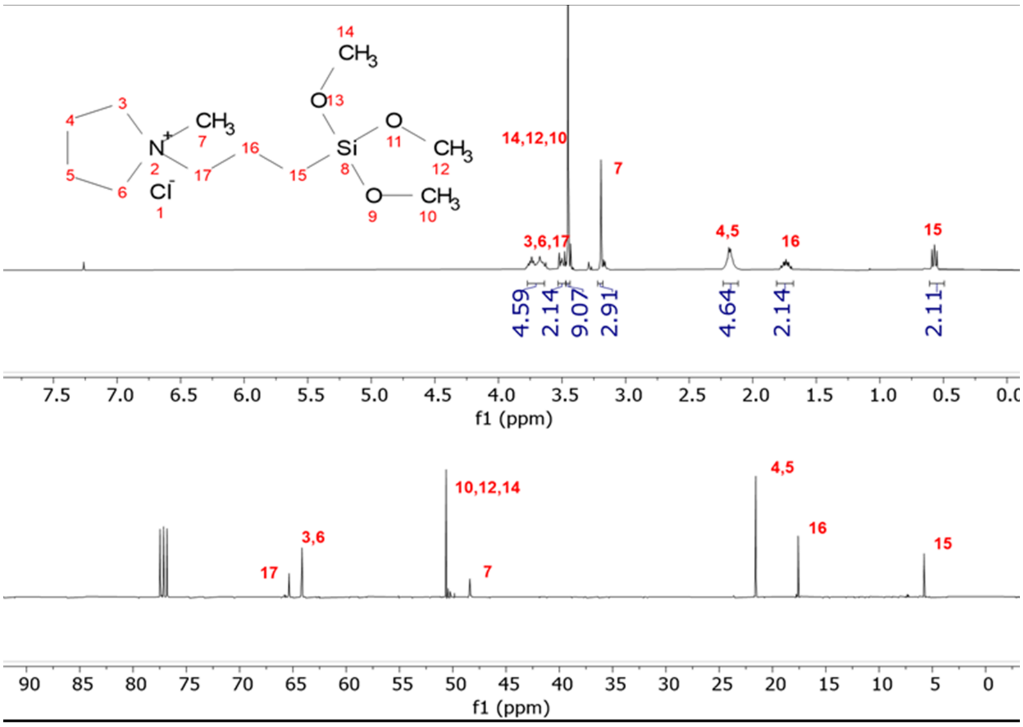

Figure S3. ^{1}H and ^{13}C NMR N-[3-(trimethoxysilyl)propyl]-N-methylpyrrolidinium chloride;

^{1}H NMR (400 MHz, Chloroform-d) δ 3.78 – 3.61 (m, 4H), 3.54 – 3.47 (m, 2H), 3.45 (s, 9H), 3.19 (s, 3H), 2.27 – 2.08 (m, 4H), 1.80 – 1.68 (m, 2H), 0.61 – 0.53 (m, 2H).

^{13}C NMR (101 MHz, Chloroform-d) δ 65.36, 64.15, 50.62, 48.40, 21.57, 17.59, 5.77

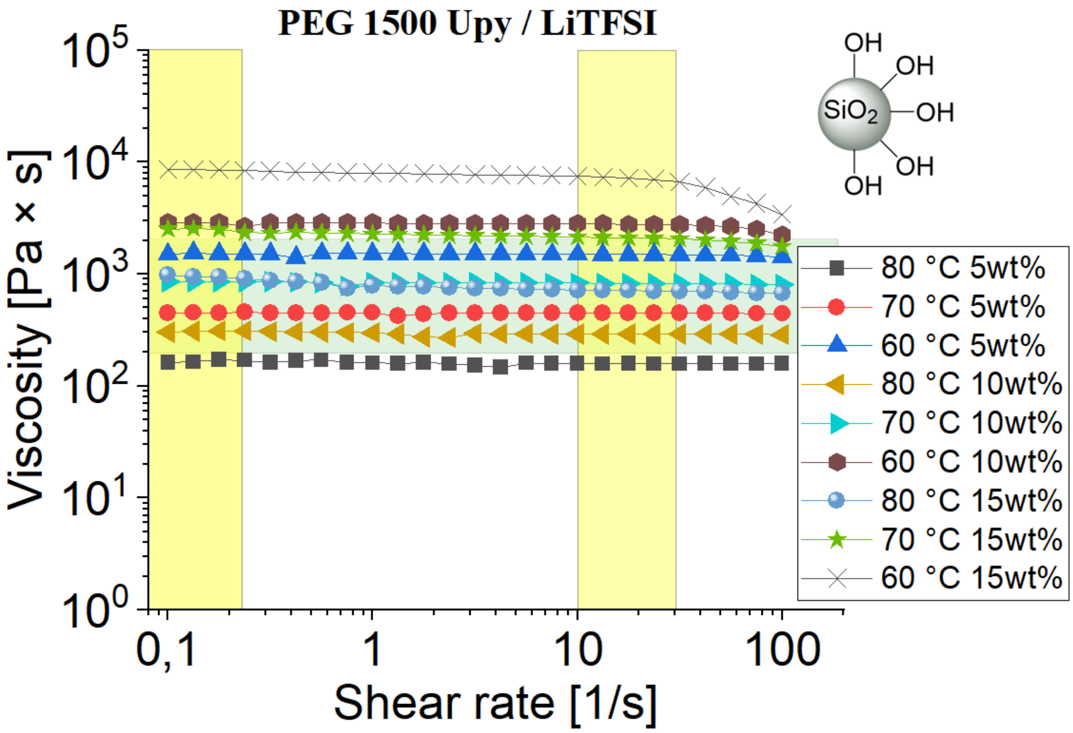

Figure S4. Rheology measurement of Viscosity vs. shear rate for PEG 1500 UPy2 mixed with NP-OH (5%, 10% 15%) 60-80°C;

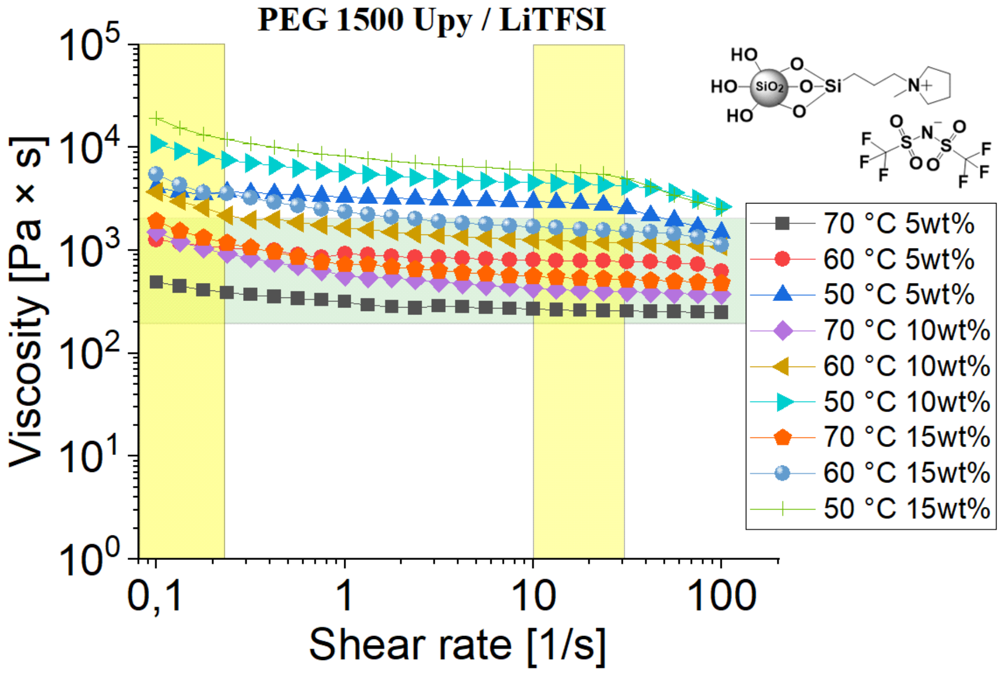

Figure S5. Rheology measurement of Viscosity vs. shear rate for PEG 1500 UPy2 mixed with NP-IL (5%, 10% 15%) 50-70°C;

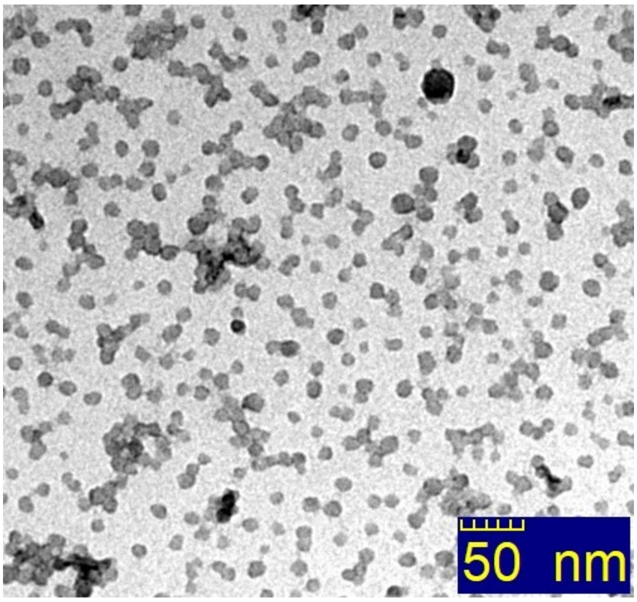

Figure S6. TEM image of NP-IL;

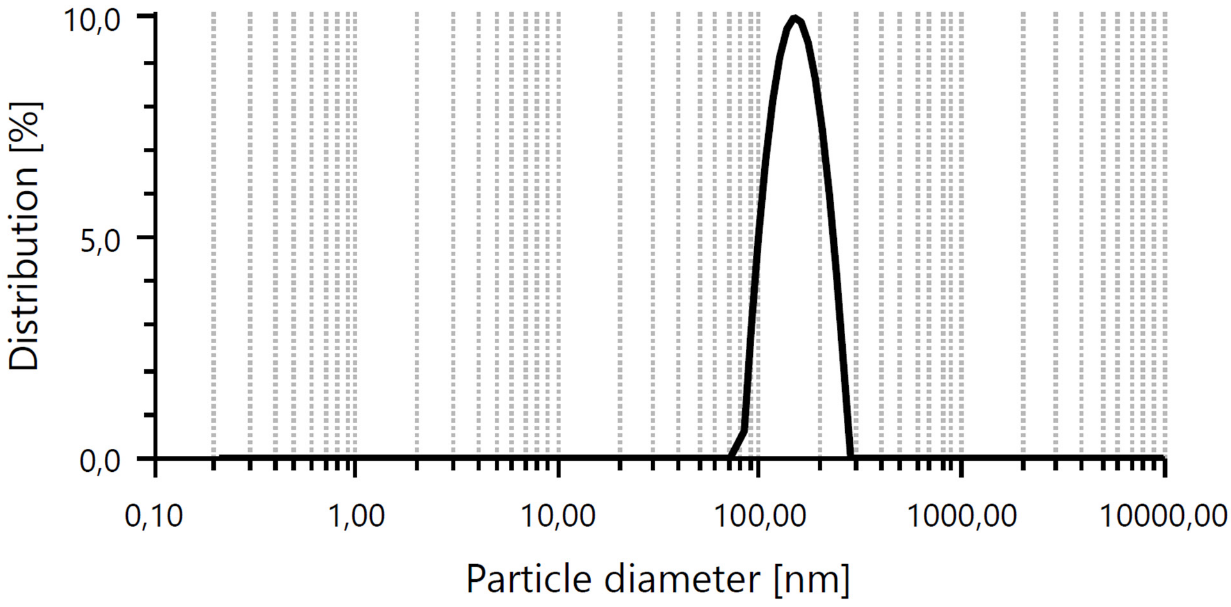

Figure S7. DLS size distribution of NP-alk;

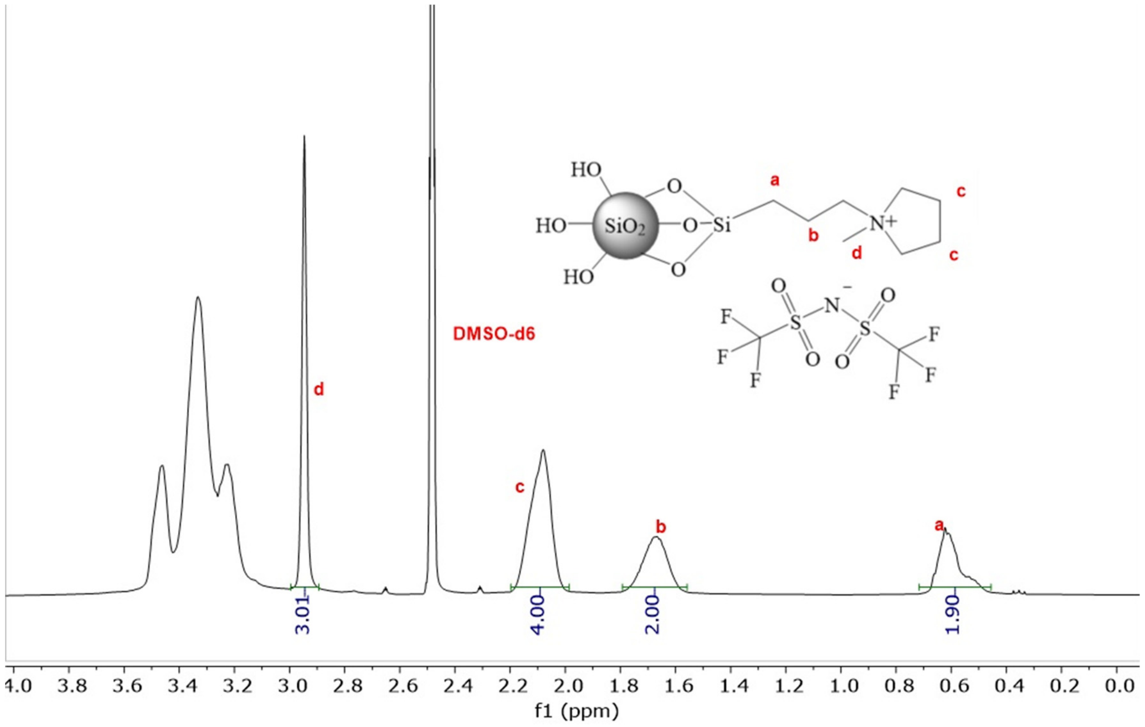

Figure S8. ^{1}H NMR of NP-IL in DMSO-d6;

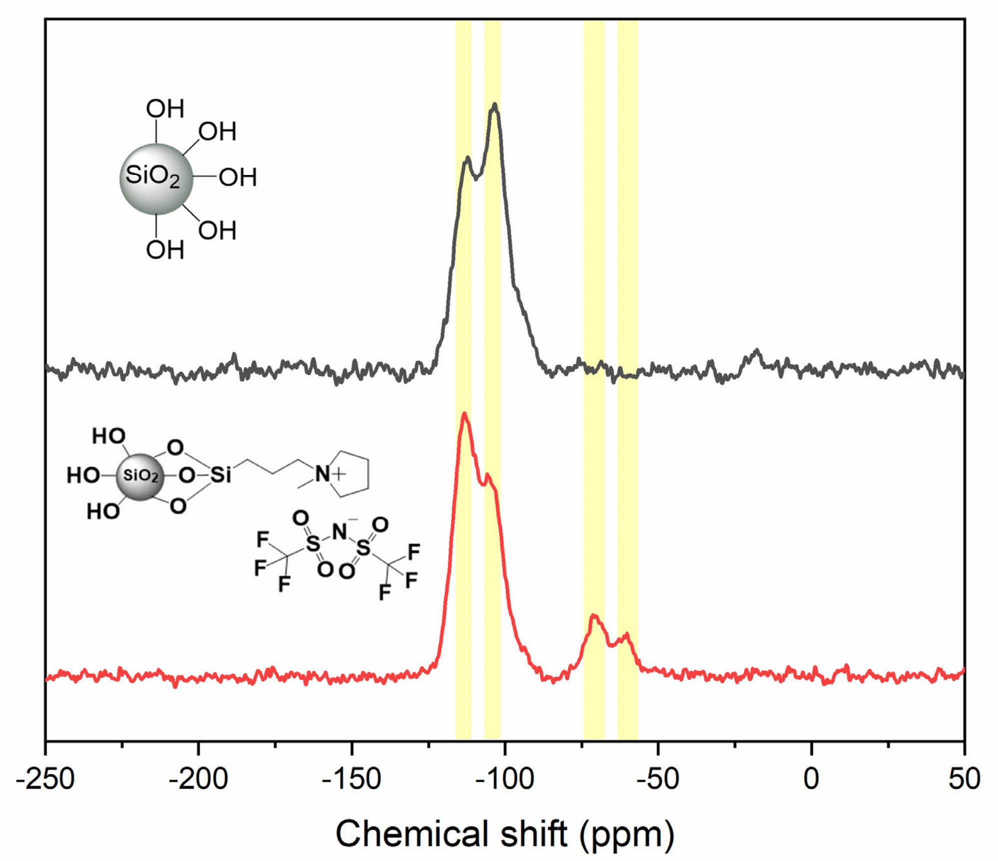

Figure S9. ^{29}Si MAS NMR spectra of NP-IL;

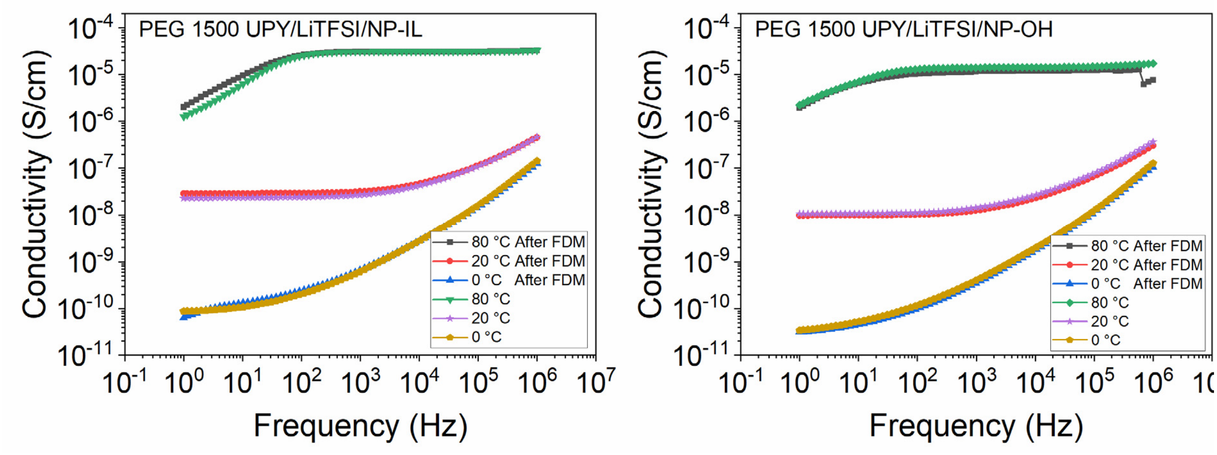

(**a**) (**b**)

Figure S10. Frequency dependent ionic conductivity of **(a)** PEG 1500 UPy / LiTFSI (EO/Li 5:1) mixed with 15wt% **NP-IL** (**4**) before and after FDM; **(b)** PEG 1500 UPy / LiTFSI (EO/Li 5:1) mixed with 15wt% **NP-OH** (**7**) before and after FDM;

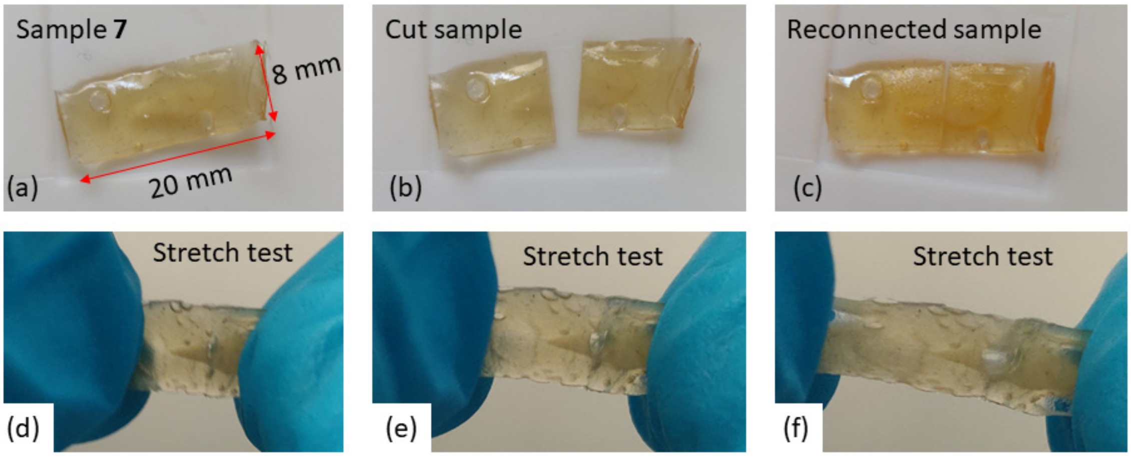

Figure S11. **(a)** PEG 1500 UPy / LiTFSI (EO/Li 5:1) mixed with 15wt% **NP-OH** (**7**); **(b)** cut sample; **(c)** Reconnected sample; **(d) (e) (f)** Stretch test after self-healing at 30 °C (in the vacuum) for 12 hours.

Instrumentation:

Nuclear Magnetic Resonance (NMR). NMR spectra were measured on a Varian Gemini 400 spectrometer (Agilent Technologies Co., USA) at 27 °C with deuterated chloroform (CDCl3) as solvent. For interpretation of the NMR spectra, MestReNova software (version 9.0.1- 13254) was utilized and chemical shift was referred by the solvent signal. Splitting patterns are designated as follows: s, singlet; d, doublet; t, triplet; and m, multiplet. Chemical shifts were given in ppm and coupling constants in Hz.

29Si MAS NMR spectra of samples were recorded on a Bruker Avance II 400 spectrometer, 4 mm MAS probe, MAS 5 kHz, with CP time of 10 ms and repetition delay of 5s. Measurements were carried out in the NMR Laboratory of the Physical Faculty at Martin-Luther University/Halle-Wittenberg in Halle, Germany. DLS measurements were conducted on Litesizer 500, Anton Paar.
Transmission electron microscopy (TEM) was performed on electron microscope EM 900 from Zeiss with an acceleration voltage of 80 kV.
